# Patient-reported outcomes after surgical treatment of spontaneous intracranial hypotension: a cross-sectional study

**DOI:** 10.1007/s13760-026-03198-3

**Published:** 2026-09-18

**Authors:** Ali Kapan, Carolina Iten, Christian T. Ulrich, Frank Donnerstag

**Affiliations:** 1 Center for Public Health, Department of Social and Preventive Medicine, Medical University of Vienna, Kinderspitalgasse 15, Vienna, 1090 Austria; 2 Department of Neurosurgery, Lindenhofspital, Bern, Switzerland; 3 Institute for Diagnostic and Interventional Neuroradiology, Hannover Medical School, Hannover, Germany

**Keywords:** Patient-Reported Outcomes, Surgery, Spontaneous Intracranial Hypotension

## Abstract

**Background:**

Spontaneous intracranial hypotension (SIH) can cause substantial functional and psychosocial impairment, but patient-reported outcomes after surgical treatment remain incompletely understood. This study aimed to describe postoperative symptom burden, functional outcomes, psychosocial impact, and headache-related disability in surgically treated patients with self-reported SIH recruited through international patient support networks.

**Methods:**

A cross-sectional online survey (February–April 2025) recruited 92 surgically treated SIH patients via international patient support networks. Variables were obtained by participant report and included comorbidities, surgical characteristics, time metrics, symptom course, psychosocial factors, and HIT-6 scores. Multivariable linear regression was performed to examine factors associated with HIT-6.

**Results:**

Median age was 42 years (IQR 38–46), and 88.0% were female. Median time from diagnosis to most recent surgery was 21 months (IQR 13–41), and time since most recent surgery was 20 months (IQR 12–36). Structural abnormalities (e.g., pseudomeningoceles, neo-membranes) were reported by 33.7% of participants. Ventral dural leaks predominated (59.8%), followed by lateral leaks (15.2%) and CSF-venous fistulae (13.0%). Complete symptom resolution was reported by 15.2% of participants. Mean HIT-6 was 56.75 (SD 12.50). In exploratory adjusted analyses, no significant associations were observed between HIT-6 scores and pre-existing headache, number of surgeries, or CSF leak type.

**Conclusion:**

In this international cross-sectional sample of surgically treated patients with self-reported SIH, a substantial proportion reported persistent symptoms, self-reported rebound intracranial hypertension, headache-related disability, and psychosocial burden. These results suggest a heterogeneous postoperative status and emphasise the importance of structured long-term follow-up.

## Introduction

Spontaneous intracranial hypotension (SIH) is a neurological disorder caused by spinal cerebrospinal fluid (CSF) leakage and can severely impact patients’ lives across multiple domains, disrupting physical, cognitive, and emotional well-being, and often leading to substantial socioeconomic consequences, including reduced work capacity, long-term sick leave, and financial strain [1, 2]. While many patients respond to conservative management such as bed rest, caffeine, and hydration, or to targeted epidural blood patching, a subset require surgical repair when these approaches fail to achieve durable leak closure. Surgical outcomes in SIH have been incompletely characterised, particularly over extended follow-up periods. A recent prospective study by Volz et al. (2023) reported a reduction in median HIT-6 scores from 65 preoperative to 49 points postoperatively; however, only 39% of the original cohort was available at the 12-month follow-up [3]. Conventional clinical follow-up studies may therefore provide an incomplete picture of long-term postoperative status. Patient advocacy networks and online support groups offer an alternative way of reaching individuals outside routine clinical follow-up, although participants recruited through these networks may differ systematically from clinic-based cohorts.

Among patients who continue to experience symptoms after dural repair, several potential mechanisms have been proposed. Incomplete closure, recurrence, or unrecognised CSF–venous fistulae may contribute to persistent CSF leakage [1, 4, 5]. Additionally, ongoing changes in intracranial compliance and glymphatic flow, along with central sensitisation resulting from prolonged intracranial hypotension, may contribute to symptom persistence [6, 7]. The heterogeneity of outcomes in conventional clinical follow-up studies poses a methodological challenge for understanding the full spectrum of long-term surgical outcomes. Given the incomplete follow-up reported in clinic-based studies, conventional cohorts may not capture the full range of postoperative experiences. Patient advocacy networks and online support groups provide an alternative means of reaching individuals outside routine clinical follow-up. However, participants recruited through these networks may differ systematically from clinic-based cohorts, and the nature and extent of these differences remain uncertain.

The primary aim of this study was to evaluate patient-reported outcomes in a sample recruited through international patient-support networks who underwent surgical intervention for SIH. Specifically, we aimed to describe the proportion of participants reporting persistent disability within this selected sample and to explore associations between clinical, surgical, or structural variables and headache-related disability measured using the HIT-6.

## Methods

### Study design and recruitment

This study was conducted using a self-report online cross-sectional survey between 1 February and 1 April 2025. The study protocol was reviewed and approved by the Ethics Committee of the Medical University of Vienna (reference number 1111/2025). Electronic informed consent was secured from all participants prior to their involvement in the survey process. The survey was conducted using the data protection-compliant platform SoSci Survey (https://www.soscisurvey.de). To prevent automated (bot) entries, reCAPTCHA verification was implemented and SoSci Survey’s duplicate protection was activated to block multiple submissions from the same device or IP address. The study was reported in accordance with the STROBE (Strengthening the Reporting of Observational Studies in Epidemiology) [8] guidelines for cross-sectional studies and the CHERRIES checklist for web-based surveys. This analysis focuses on individuals who underwent surgical treatment for SIH [9]. To recruit participants internationally, the survey link was distributed via established digital channels within the global intracranial hypotension (IH) community, including patient organisations such as the Spinal CSF Leak Foundation (USA), the CSF Leak Association (UK), CSF Leak Canada, the Spanish-speaking Asociación AHIFUGA, and several international Facebook support groups. These communities facilitate experience sharing and provide emotional support and guidance throughout often lengthy diagnostic and therapeutic processes.

### Study population

Participants were recruited as part of a broader study including both SIH and iatrogenic CSF leaks. The present manuscript focuses exclusively on the SIH cohort. The inclusion and exclusion criteria were clearly defined in all recruitment materials, such as online support group postings. Participants explicitly confirmed their eligibility at the beginning of the questionnaire. The survey used filter logic to automatically exclude respondents who did not meet the eligibility criteria. Incomplete responses were also excluded from the final analyses, particularly from individuals who only completed the demographic questionnaires. Additionally, participants were asked to refer to their medical reports or physician letters when answering questions related to clinical characteristics and diagnostic findings. Eligible participants included individuals who were at least 18 years old and answered “yes” to the screening question: “Have you received surgery for a cerebrospinal fluid (CSF) leak?” Participants were then asked to specify whether their CSF leak was spontaneous (SIH) or iatrogenic. Only individuals reporting spontaneous intracranial hypotension were included in the present analysis. Other inclusion criteria comprised sufficient English language proficiency and electronically submitted informed consent. All clinical and surgical variables, including CSF leak type and structural findings, were self-reported. The participant information sheet informed participants that the questionnaire might cause emotional distress and recommended that they seek support from local organisations if necessary. As the responses were collected anonymously, it was not possible to provide individual follow-up based on the responses to the questionnaire.

### Questionnaire development and patient-centred co-design

The questionnaire was developed specifically for this study in collaboration with patient representatives and administrators of patient support communities. This participatory approach was intended to improve the relevance, practicality, and comprehensibility of the questionnaire for the target population. The involvement of affected individuals in research aligns with current recommendations for Patient and Public Involvement and was intended to improve the relevance and comprehensibility of the questionnaire. To ensure high data quality, automatic completeness checks were implemented [10]. 

### Questionnaire structure

The full version of the questionnaire is provided as Supplementary File 1. The questionnaire comprised the following domains:

- Sociodemographic characteristics: age, gender, body measurements, marital status, education, employment, and income.
- Clinical characteristics: spontaneous CSF leak characteristics, prior spinal procedures, history of primary headache disorders (e.g. migraine, tension-type headache), pre-onset headache frequency, and relevant comorbidities (e.g. Ehlers–Danlos syndrome, POTS, ME/CFS).
- Medical treatment and course: surgical interventions (time from SIH diagnosis to most recent surgery, number of surgeries, spinal levels, leak location and type, and time elapsed since most recent surgery**)**, postoperative complications, and subjectively perceived treatment effectiveness.
- Functional and occupational consequences: changes in physical activity, return to work or medical retirement, and postoperative limitations in daily functioning.
- Psychosocial impact: psychological symptoms, use of psychological support, emotional distress, and effects on close relationships.

### Headache-related disability

The Headache Impact Test–6 (HIT-6) was employed to assess headache-related disability. The HIT-6 is a standardised, validated instrument for evaluating daily life impairment caused by headache. It consists of six items addressing pain intensity, social function, cognitive performance, and emotional burden. Total scores range from 36 to 78 points, with higher values indicating greater impairment. Four disability categories are typically distinguished: little or no impact (≤ 49), moderate impact (50–55), substantial impact (56–59), and severe disability (≥ 60) [11]. 

### Sample size

No formal a priori sample size calculation was performed due to the lack of reliable data to inform effect size estimation for multivariable modelling in surgically treated SIH populations. Consequently, an exploratory study design was chosen, and all available data were included in the analysis.

### Statistics

Continuous variables were assessed for distribution visually and using the Shapiro–Wilk test. As most variables were non-normally distributed, they are reported as medians with interquartile ranges (IQR). Categorical variables are presented as frequencies and percentages.

Given the exploratory, cross-sectional design and the limited sample size, multivariable modelling was restricted to a small number of clinically selected variables. A linear regression analysis was performed with the Headache Impact Test–6 (HIT-6) total score as the dependent variable. The main explanatory variables were pre-existing headache, number of surgeries, and CSF leak type. Participants with an unknown CSF leak type were excluded from the respective analysis.

In Model 1, each explanatory variable was analysed separately and adjusted for age, sex, and time since the most recent surgery. Model 2 included all listed explanatory variables simultaneously and was adjusted for age, sex, and time since the most recent surgery. The assumptions of linear regression were assessed by visual inspection of histograms and normal P–P plots of the standardised residuals, plots of standardised residuals against standardised predicted values to assess homoscedasticity, and Cook’s distance to identify influential observations. Associations are reported as β coefficients with 95% confidence intervals (CI). Given the exploratory nature of the study, p-values were interpreted descriptively and no adjustment for multiple comparisons was applied. All analyses were conducted using SPSS version 28.0 (IBM Corp., Armonk, NY).

## Results

The online survey received 582 clicks, and 302 individuals initiated the questionnaire. Of these, 27 were excluded because they had not undergone surgery and 21 because they reported skull base CSF leaks, leaving 254 eligible respondents. A further 26 withdrew after the inclusion questions or provided incomplete responses, yielding a final sample of 228 participants. Of these, 92 (40.4%) self-reported spontaneous intracranial hypotension (SIH) and 136 (59.6%) self-reported iatrogenic CSF leaks.

Sociodemographic and clinical characteristics of the SIH cohort are presented in Table 1. The median age was 42 years (IQR 38–46), and 81 participants (88.0%) were female. Fifty-one participants (55.4%) were employed at the time of the survey, whereas 16 (17.4%) were medically retired due to their CSF leak. Ten individuals (10.9%) were unemployed. With respect to medication use, 62 patients (67.4%) reported treatment with tricyclic antidepressants, 43 (46.7%) with acetazolamide, and 30 (32.6%) with gabapentin or pregabalin. Beta blockers were used by 14 participants (15.2%).

**Table 1:** Sociodemographic and Clinical Characteristics of Individuals with Spontaneous Intracranial Hypotension

Characteristic	Total (*n* = 92)
Age (years), median (IQR)	42 (38–46)
**Sex**,** n (%)**	
Female	81 (88.0)
Body weight (kg), median (IQR)	65 (60–77)
Height (cm), median (IQR)	168 (164–173)
**Marital Status**,** n (%)**	
Married	24 (26.1)
Divorced	10 (10.9)
Single	31 (33.7)
Widowed	0
In a relationship	27 (29.3)
Prefer not to answer	0
**Educational Attainment**,** n (%)**	
Less than high school	1 (1.1)
High school or some college	37 (40.2)
Bachelor’s degree	26 (28.3)
Graduate degree	28 (30.4)
**Monthly net income**,** n (%)**	
< €1500	15 (16.3)
€1500–€2999	26 (28.3)
€3000–€4999	17 (18.5)
≥ €5000	12 (13.0)
Prefer not to answer	22 (23.9)
**Employment Status**,** n (%)**	
Training/apprenticeship	3 (3.3)
Employed	51 (55.4)
Unemployed	10 (10.9)
Retired (age-related)	0
Medically retired (due to CSF leak)	16 (17.4)
Self-employed	8 (8.7)
Student	2 (2.2)
Prefer not to answer	2 (2.2)
**Postoperative medication use**,** n (%)**	
Acetazolamide	43 (46.7)
Gabapentin/Pregabalin	30 (32.6)
Theophylline	20 (21.7)
Tricyclic antidepressants	62 (67.4)
Beta blockers	14 (15.2)

Note: Values are shown as median (IQR) for continuous variables and as counts (percentage) for categorical variables. Participants could report more than one medication. Abbreviations: CSF = cerebrospinal fluid; SIH = spontaneous intracranial hypotension; kg = kilogram; cm = centimetre; € = Euro

Pre-existing headache characteristics and comorbidities are shown in Table 2. Most participants (76.1%) reported no pre-existing headache prior to the onset of SIH; migraine (9.8%) and tension-type headache (8.7%) were the most common headache disorders among the remainder. Pre-onset headache frequency was generally low.

**Table 2:** Clinical Characteristics and Comorbidities in Surgically Treated SIH Patients

Variables	Total (*n* = 92)
**Pre-existing Headache Type before onset of SIH, n (%)**	
Migraine	9 (9.8)
Tension-type headache	8 (8.7)
Undiagnosed headache	4 (4.3)
Cluster headache	1 (1.1)
No pre-existing headache	70 (76.1)
**Pre-existing Headache Frequency**,** n (%)**	
Less than one day per month	10 (10.9)
One to 14 days per month	6 (6.5)
15 or more days per month	7 (7.6)
**Comorbidities**,** n (%)**	
Ehlers-Danlos syndrome	9 (9.8)
Postural orthostatic tachycardia syndrome	5 (5.4)
Chiari malformation	5 (5.4)
Fibromyalgia	5 (5.4)
Syringomyelia	4 (4.3)
ME/CFS	3 (3.3)
Malignancy	3 (3.3)
Mast cell activation syndrome	2 (2.2)
Superficial siderosis	2 (2.2)
Scleroderma	2 (2.2)
Stroke	2 (2.2)
Adhesive arachnoiditis	1 (1.1)
Multiple sclerosis	1 (1.1)

Note: Values are presented as absolute numbers (percentages). Participants could report more than one comorbidity or type of headache. SIH = spontaneous intracranial hypotension; ME/CFS = myalgic encephalomyelitis/chronic fatigue syndrome

Ehlers–Danlos syndrome was present in 9.8% of patients. Postural orthostatic tachycardia syndrome, Chiari malformation, and fibromyalgia were each reported in 5.4% of cases. All other comorbidities were observed in fewer than 5% of participants.

The surgical characteristics are summarised in Table 3. The median time from diagnosis to most recent CSF leak surgery was 21 months (IQR 13–41), and the median time since surgery was 20 months (IQR 12–36, range 2–120 months). Most participants (72.8%) reported that surgery was performed at a specialised CSF leak centre. Overall, 63.0% underwent one surgery, whereas 37.0% required two or more procedures. Leak location was most frequently reported in the thoracic spine (56.5%), followed by the lumbar spine (35.9%), with cervical and sacral locations being less common. Ventral dural CSF leaks predominated (59.8%), followed by lateral leaks (15.2%) and CSF-venous fistulae (13.0%). Structural abnormalities were reported by 33.7% of participants, while 48.9% reported no structural findings.

**Table 3:** Surgical Profiles and Complications in Surgically Treated SIH Patients

Variables	Total (*n* = 92)	
**Surgical Treatment Characteristics, median (IQR)**		
Time from diagnosis to most recent CSF leak surgery (months)	21 (13–41)	
Elapsed time since CSF leak surgery (months)	20 (12–36)	
Sick leave duration since CSF leak surgery (months)	9 (4–15)	
**Surgery performed at a specialized CSF leak center**,** n (%)**		
Yes	67 (72.8)	
No	24 (26.1)	
I don’t know	1 (1.1)	
**Number of CSF leak surgeries**,** n (%)**		
1 surgery	58 (63.0)	
2 surgeries	21 (22.8)	
3 surgeries	7 (7.6)	
4 surgeries	3 (3.3)	
5 surgeries	3 (3.3)	
**CSF leak location**,** n (%)**		
Neck (cervical spine)	5 (5.4)	
Upper back (thoracic spine)	52 (56.5)	
Lower back (lumbar spine)	33 (35.9)	
Sacral	2 (2.2)	
**Type of spinal CSF leak**,** n (%)**		
Ventral dural CSF leak	55 (59.8)	
Dorsal dural CSF leak	3 (3.3)	
Lateral CSF leak	14 (15.2)	
CSF-venous fistula	12 (13.0)	
Unknown	8 (8.7)	
**Structural findings**,** n (%)**		
Combined pseudomeningocele/epidural neomembranes	10 (10.9)	
Pseudomeningocele	13 (14.1)	
Neomembrane	8 (8.7)	
I don’t know	16 (17.4)	
No structural findings	45 (48.9)	

Note: Values are presented as absolute numbers (percentages) or median (IQR). All participants had taken sick leave after surgery (*n* = 92). CSF = cerebrospinal fluid; SIH = spontaneous intracranial hypotension; IQR = interquartile range

Postoperative outcomes are presented in Table 4. Complete symptom resolution was reported by 15.2% of participants, and 39.1% reported significant sustained improvement. Infections (42.4%) and sleep disturbances (37.0%) were the most frequently reported symptom-exacerbating factors, followed by stress or emotional state (34.8%) and physical activity (31.5%). Regarding postoperative headache phenotype, 46.8% described a self-reported high-pressure headache phenotype, while 15.2% reported persistent orthostatic headache. Diffuse non-specific headache was reported by 19.5%, and 17 participants (18.5%) reported complete headache resolution. Return to work without restrictions was achieved by 56.5%, whereas 19.6% remained on medical leave and 8.7% were medically retired due to chronic CSF leak-related symptoms.

**Table 4:** Postoperative Symptom Progression, Physical Activity, and Occupational Outcomes in Surgically Treated SIH Patients

Variables	SIH (*n* = 92)
**Post-surgical symptom trajectory, n (%)**	
Complete symptom resolution	14 (15.2)
Significant sustained improvement	36 (39.1)
Mild improvement with fluctuations	20 (21.7)
No change in symptom severity	8 (8.7)
Initial improvement with subsequent relapse	10 (10.9)
Symptom worsening since surgery	3 (3.3)
**Symptom exacerbating factors**,** n (%)**	
Weather changes	31 (33.7)
Physical activity	29 (31.5)
Stress or emotional state	32 (34.8)
Sleep quality	34 (37.0)
Infections	39 (42.4)
Sodium intake	19 (20.7)
Hormonal changes (menstrual cycle)	15 (16.3)
**Headache Type Post-Surgery**,** n (%)**	
Persistent orthostatic headache (low-pressure phenotype)	14 (15.2)
Rebound intracranial hypertension (high-pressure phenotype)	43 (46.8)
Diffuse, non-specific headache	18 (19.5)
Complete resolution (no postoperative headache)	17 (18.5)
**Employment Status after CSF Leak Surgery**,** n (%)**	
Returned to work without changes (same hours, same position)	52 (56.5)
Currently on medical leave since surgery	18 (19.6)
Working with reduced hours due to persistent symptoms	7 (7.6)
Medically retired due to chronic CSF leak	8 (8.7)
Unemployed, but not due to health reasons	7 (7.6)
Retired due to age (not health-related)	0
**Change in physical activity levels since surgery**,** n (%)**	
Marked increase in activity level	39 (42.4)
Slight increase in activity level	20 (21.7)
No change in activity level	26 (28.3)
Decrease in activity level	7 (7.6)
**Types of physical activity currently possible since surgery**,** n (%)**	
Walking (leisure)	60 (65.2)
Cycling	20 (21.7)
Swimming	26 (28.3)
Yoga or pilates	12 (13.0)
Strength or resistance training	18 (19.6)
Team sports (e.g., soccer, basketball)	2 (2.2)
Fear of being physically active after surgery	25 (27.2)
Complete inability to engage in physical activity	10 (10.9)

Note: Values are shown as absolute numbers (percentages). Respondents could choose multiple factors that exacerbate symptoms and various types of physical activity. The category “persistent orthostatic headache” refers to ongoing symptoms despite surgery, while “resolution” indicates complete disappearance of orthostatic symptoms after surgery. CSF = cerebrospinal fluid; SIH = spontaneous intracranial hypotension

Psychosocial outcomes and headache-related disability are shown in Table 5. A substantial proportion of participants reported emotional distress following SIH onset: 60.9% described significant psychological strain, and 37.0% reported feeling emotionally affected. Regarding psychological support, 33.7% were currently receiving therapy and 31.5% had received therapy previously, while 17.4% indicated that support would be helpful but had not yet accessed it. Frequent anxiety or fear was the most commonly reported current psychological symptom (52.2%), followed by difficulty coping with daily responsibilities (41.3%) and persistent sadness or hopelessness (39.1%). Nearly one-fifth (18.5%) reported recurrent thoughts of self-harm or suicidal ideation. The mean HIT-6 score was 56.75 (SD 12.50), with 27.2% of participants falling into the severe disability category.

**Table 5:** Psychosocial Outcomes and Headache Impact in Surgically Treated SIH Patients

Characteristic	Total *n* = 92
**Relationship changes since onset of SIH, n (%)**	
Yes, relationship was strained but remained intact	24 (26.1)
Yes, ongoing relationship difficulties persist	32 (34.8)
Yes, caused significant issues and led to separation/divorce	6 (6.5)
No, no significant impact on relationship	22 (23.9)
Prefer not to answer	8 (8.7)
**Do you believe that SIH has caused emotional distress? n (%)**	
Yes, I experience significant emotional distress with psychological strain	56 (60.9)
Yes, I feel emotionally affected, but I am unsure whether it constitutes significant distress	34 (37.0)
No, I do not experience emotional distress	2 (2.2)
Prefer not to answer	0
**Use of psychological support since onset of SIH**,** n (%)**	
Yes, currently receiving psychological therapy	31 (33.7)
Yes, previously received psychological therapy	29 (31.5)
No, but I believe psychological support would be helpful	16 (17.4)
No, I do not believe psychological support is necessary	13 (14.1)
No, I have not considered psychological support	3 (3.3)
**Current psychological symptoms**,** n (%)**	
Persistent sadness or hopelessness	36 (39.1)
Frequent anxiety or fear	48 (52.2)
Difficulty coping with everyday responsibilities or stressors	38 (41.3)
Recurrent thoughts of self-harm or suicide	17 (18.5)
Prefer not to answer	0
**Headache Impact Test-6**,** mean (SD)**	56.75 (12.50)
**HIT-6 headache-related disability categories**,** n (%)**	
Little or no headache-related impact (≤ 49)	21 (22.8)
Moderate headache-related impact (50–55)	26 (28.3)
Substantial headache-related impact (56–59)	20 (21.7)
Severe headache-related disability (≥ 60)	25 (27.2)

Note: All values are shown as absolute numbers (percentages) unless otherwise specified. Respondents could report multiple psychological symptoms. Emotional distress and the use of psychological support were self-reported. SIH = spontaneous intracranial hypotension; CSF = cerebrospinal fluid; HIT-6 = Headache Impact Test–6; SD = standard deviation

In exploratory adjusted linear regression analyses, no statistically significant associations were observed between HIT-6 scores and pre-existing headache, number of surgeries, or CSF leak type in either Model 1 or Model 2 (Table 6). Visual inspection of the residual diagnostics indicated no substantial violations of normality or homoscedasticity, and Cook’s distances did not identify influential observations.

**Table 6:** Multivariable Linear Regression Analysis of Factors Associated with HIT-6 Score in Surgically Treated SIH Patients

Model	Variable	β (95% CI)	*p*-value
1a (*n* = 92)	Pre-existing headache: Yes vs. No	−3.47 (− 8.87 to 1.92)	0.204
	Age	−0.08 (− 0.45 to 0.30)	0.685
	Female vs. Male	0.28 (− 8.20 to 8.75)	0.948
	Time since surgery	0.02 (− 0.08 to 0.11)	0.757
1b (*n* = 92)	Number of surgeries	−1.24 (− 3.90 to 1.42)	0.357
	Age	−0.10 (− 0.47 to 0.28)	0.610
	Female vs. Male	−0.68 (− 9.18 to 7.82)	0.874
	Time since surgery	0.00 (− 0.09 to 0.10)	0.982
1c (*n* = 84)	Ventral vs. CSF-venous fistula	3.78 (− 5.17 to 12.72)	0.403
	Dorsal vs. CSF-venous fistula	4.80 (− 4.14 to 13.75)	0.288
	Lateral vs. CSF-venous fistula	−2.52 (− 17.69 to 12.66)	0.742
	Age	−0.03 (− 0.45 to 0.40)	0.903
	Female vs. Male	0.26 (− 9.30 to 9.82)	0.957
	Time since surgery	0.01 (− 0.10 to 0.11)	0.904
2 (*n* = 84)	Pre-existing headache: Yes vs. No	−2.91 (− 9.12 to 3.31)	0.354
	Number of surgeries	−1.12 (− 3.96 to 1.72)	0.436
	Ventral vs. CSF-venous fistula	2.60 (− 6.60 to 11.80)	0.575
	Dorsal vs. CSF-venous fistula	4.37 (− 4.63 to 13.37)	0.336
	Lateral vs. CSF-venous fistula	−2.41 (− 17.66 to 12.85)	0.754
	Age	−0.05 (− 0.48 to 0.37)	0.808
	Female vs. Male	0.06 (− 9.63 to 9.74)	0.990
	Time since surgery	0.02 (− 0.09 to 0.12)	0.763

Note: A linear regression analysis was performed with the Headache Impact Test–6 (HIT-6) total score as the dependent variable. Model 1a included pre-existing headache, Model 1b number of surgeries, and Model 1c CSF leak type; each model was adjusted for age, sex, and time since the most recent surgery. Model 2 included all explanatory variables simultaneously and the same adjustment covariates. The reference categories were no pre-existing headache, male sex, and CSF-venous fistula. Model fit was as follows: Model 1a: R² = 0.021, adjusted R² = −0.024; Model 1b: R² = 0.012, adjusted R² = −0.034; Model 1c: R² = 0.026, adjusted R² = −0.051; Model 2: R² = 0.047, adjusted R² = −0.056. β = unstandardised regression coefficient; CI = confidence interval; CSF = cerebrospinal fluid; HIT-6 = Headache Impact Test–6; SIH = spontaneous intracranial hypotension

## Discussion

This study describes the postoperative condition of individuals with self-reported SIH recruited through international patient support networks. Functional and occupational limitations were common. The median duration of sick leave was nine months (IQR 4–15). At the time of the survey, 56.5% of participants had returned to work without changes; 19.6% were still on medical leave; 8.7% had been medically retired due to symptoms related to a CSF leak; and 7.6% were working reduced hours due to persistent symptoms. These figures suggest that postoperative status did not equate to full occupational recovery for all participants in this self-selected cohort. Most participants continued to report symptoms at the time of the survey. Complete resolution of all symptoms was reported by 15.2% of participants, while the majority described persistent, incomplete, or fluctuating symptoms. Headache symptoms persisted in most participants, most frequently presenting as a self-reported high-pressure headache phenotype (46.8%), while 15.2% reported persistent orthostatic low-pressure headache. As the HIT-6 captures current headache impact, these ongoing symptoms are consistent with the relatively high HIT-6 scores in our sample.

For comparison, Volz et al. (2023) reported a reduction in median HIT-6 scores from 65 preoperatively to 49 postoperatively [3]. In our support-network sample, the mean HIT-6 score was 56.75 (SD 12.50), with 48.9% of participants classified as having substantial or severe headache-related disability. As no preoperative data were available and recruitment strategies differed, direct comparisons are not possible. Nevertheless, the present cohort shares several characteristics with prospective clinic-based SIH series, including a predominance of female participants (88.0%), a median age of 42 years, and a comparable proportion of ventral leaks (approximately 60%). The median self-reported interval from diagnosis to the most recent surgery was 21 months. For context, Volz et al. (2024) reported a median symptom duration of 5 months before surgery, and Häni et al. (2022) reported a mean duration of approximately 12 months [1, 6]. Häni et al. (2022) also reported complete symptom resolution in 71.4% of patients treated within 12.4 weeks of symptom onset, compared with 37.5% of those treated later [1]. Volz et al. (2024) similarly observed greater HIT-6 improvement in patients with a symptom duration below 90 days, although the postoperative difference was not statistically significant [6]. These clinic-based findings suggest that the timing of treatment may be clinically relevant for patients with SIH. However, the reported intervals are not directly comparable, as our study measured the time elapsed between diagnosis and the most recent surgery, rather than the time elapsed between symptom onset and treatment. Consequently, our data do not permit a direct assessment of whether earlier surgical treatment is associated with lower postoperative HIT-6 scores. More broadly, given the modest sample size, the regression models were deliberately restricted to a small number of clinically selected explanatory variables to reduce the risk of overfitting. Nevertheless, the limited sample size and wide confidence intervals may have reduced the ability to detect clinically meaningful associations. Accordingly, non-significant findings should not be interpreted as evidence that the examined variables are clinically unimportant.

Beyond functional and occupational limitations, participants reported a considerable psychological burden. Some degree of emotional distress was reported by 97.9%, and 33.7% were receiving psychological therapy at the time of the survey. Frequent anxiety or fear was reported by 52.2% of participants, difficulty coping with everyday responsibilities by 41.3%, and persistent sadness or hopelessness by 39.1%. Recurrent thoughts of self-harm or suicide were reported by 18.5% of participants. As these outcomes were assessed using self-reported survey questions rather than validated psychiatric instruments or formal clinical assessments, they should be interpreted as patient-reported psychological symptoms rather than psychiatric diagnoses. These findings are consistent with previous evidence of psychological distress among patients with SIH. Liaw et al. (2023) reported high levels of depression, anxiety, and suicidality in a non-surgical SIH cohort [12]. Volz et al. (2024) assessed a surgical cohort using the validated PHQ-8 and found clinically relevant depressive symptoms (PHQ-8 ≥ 10) in 63.2% of patients before surgery and 21.4% at the six-month follow-up [13]. While differences in study populations and assessment methods prevent direct comparison, these studies suggest that psychological symptoms are an important component of the patient-reported burden of SIH. Participants also reported substantial social burden. Ongoing relationship difficulties were reported by 34.8% of participants, while 6.5% reported illness-related separation or divorce. These findings are consistent with those of Jesse et al. (2022), who reported a severe impact on partnerships in 31.6% of patients, divorce related to the illness in 6.3%, and a continuing moderate or severe impact on social life in 41.5% [14]. Together, these findings indicate that the patient-reported burden in this sample extended beyond headaches and occupational limitations to include psychological well-being, close relationships, and social participation.

### Limitations

This study has several limitations. Its cross-sectional design precludes causal inference and does not permit assessment of surgical effectiveness or recovery trajectories. In addition, the absence of preoperative baseline measures (e.g., HIT-6) prevents assessment of changes in headache burden following surgery. Prospective longitudinal studies with repeated assessments are required to evaluate postoperative changes over time. Recruitment exclusively through patient organisations and online support groups likely introduced selection bias, as individuals with persistent symptoms, recurrent disease, or ongoing healthcare needs may have been more likely to participate, whereas fully recovered patients may have been underrepresented. Consequently, this sample should not be considered representative of all surgically treated patients with SIH, and the reported symptom burden may be overestimated. The study was further limited to individuals who had undergone surgical intervention, and the findings may not generalise to patients with SIH managed conservatively or with epidural blood patching alone. The use of an English-language questionnaire may further limit generalisability by excluding non-English-speaking patients, and we did not record participants’ native language or whether translation or disability-related assistance was used in completing the survey.

All clinical and anatomical variables, including comorbidities, CSF leak type, CSF-venous fistulae, pseudomeningoceles, neomembranes, and surgical details, were self-reported and could not be independently verified or further characterised. Although participants were asked to consult their medical records, misclassification cannot be excluded. Analyses involving self-reported clinical and anatomical variables were exploratory and should therefore be interpreted with caution. Finally, although the regression models were deliberately restricted to a small number of clinically selected explanatory variables to reduce the risk of overfitting, the modest sample size and wide confidence intervals may have limited the ability to detect clinically meaningful associations.

## Conclusion

Among surgically treated participants with self-reported SIH recruited through international patient support networks, a substantial proportion reported headache-related disability and psychosocial burden. Exploratory adjusted analyses did not identify significant associations between HIT-6 scores and selected clinical variables. These findings indicate heterogeneous postoperative status within this selected population and underscore the importance of structured long-term follow-up pathways.

## Data Availability

The datasets generated and analysed during the current study are not publicly available due to data protection regulations and the presence of sensitive patient-reported health data. Data may be available from the corresponding author on reasonable request, subject to applicable ethical and data protection requirements.
